# The Relation Between College Students’ Social Anxiety and Mobile Phone Addiction: The Mediating Role of Regulatory Emotional Self-Efficacy and Subjective Well-Being

**DOI:** 10.3389/fpsyg.2022.861527

**Published:** 2022-05-02

**Authors:** Zhenlei Xiao, Jianhao Huang

**Affiliations:** ^1^Hengshui University, Hengshui, China; ^2^Dhurakij Pundit University, Bangkok, Thailand

**Keywords:** social anxiety, regulatory emotional self-efficacy, subjective well-being, mobile phone addiction, college students

## Abstract

The present study explores the underlying mechanism of the relationship between college students’ social anxiety and mobile phone addiction. Adopting college students’ social anxiety scale, regulatory emotional self-efficacy scale, subjective well-being scale and mobile phone addiction scale, this research tested valid samples of 680 Chinese college students. The results indicated that social anxiety exerted a significant and positive impact on mobile phone addiction. Regulatory emotional self-efficacy played a partial mediating role between social anxiety and mobile phone addiction. Subjective well-being also played a partial mediating role between social anxiety and mobile phone addiction. Moreover, both regulatory emotional self-efficacy and subjective well-being were found to play a chain mediating role between social anxiety and mobile phone addiction. The study provides valuable insights into the impact of college students’ social anxiety on mobile phone addiction.

## Introduction

The advent of smartphones has remarkably changed people’s living standards and made our life convenient; however, the simultaneously aroused problem of mobile phone addiction cannot be ignored. Addiction to mobile phones, particularly among teenagers, has become a vital social issue (Caprara et al., 2002; Lian, 2017; Elhai et al., 2018; Zsido et al., 2021). This newly emerged addiction behavior is associated with a series of mental and behavioral problems among individuals because of their excessive dependence on and usage of mobile phones (Lapierre et al., 2019). College students are open-minded, display good social adaptability, and are flexible at embracing new technology. However, they have more free time, and thus, they treat mobile phones as their personal friends. Consequently, college students are addicted to various mobile phone functions, and their addiction behavior is particularly serious (Kim and Koh, 2018; Yu et al., 2021).

Davis (2001) asserted that the individual susceptible qualities such as depression, social anxiety, and material dependence are the root causes of Internet addiction. Numerous studies conducted on the possible factors for mobile phone addiction suggest that social anxiety is one of the most direct factors leading to mobile phone addiction (Kong et al., 2020). According to social psychology, the long-term use of mobile phones to communicate with people significantly increases the probability of personal sociability decline, and the lack of normal social communication can increase the probability of loneliness and emptiness; thus, mobile phone addiction possibly contributes to the development of negative emotions (Maya and Nazir, 2016; Liu et al., 2017; Zhang et al., 2018). Social anxiety is a psychosocial problem, and people with social anxiety prefer other forms of communication, rather than face-to-face communication, particularly through mobile technology devices because this mode of communication can reduce anxiety (Darcin et al., 2016).

Individuals with social anxiety exhibit emotional instability, impatience, and impulsivity; express their emotions through repression and concealing (Wu and Liu, 2006); and display poor emotion regulation ability and low self-efficacy (Kashdan et al., 2011). Regulatory emotional self-efficacy, a type of self-efficacy, not only has a direct impact on behaviors but also can indirectly affect behaviors by influencing cognition, motivations, decisions and emotions, thereby playing a pivotal role in regulating individual personality and behavior and further maintaining the mental health state of individuals (Tang et al., 2010). The impact of emotions on health and behavior is mediated by regulatory emotional self-efficacy. Furthermore, it enables individuals to effectively cope with stress, improve their subjective well-being, and also plays a crucial role in addiction behavior (Bandura et al., 2003). Individuals with high regulatory emotional self-efficacy can actively change their environment to better deal with various problems under adverse situations, which may improve their subjective well-being (Huang et al., 2016). Teenagers who are dissatisfied with their well-being in real life achieve satisfaction by indulging in the Internet. According to a study, the individual Internet addiction and subjective well-being are significantly correlated (Akin, 2012). Given the aforementioned facts, the present research aims to explore the effect of social anxiety on college students’ mobile phone addiction behavior, as well as the mediating role of regulatory emotional self-efficacy and subjective well-being. Knowledge of the underlying mechanism behind the correlation between social anxiety and college students’ mobile phone addiction behavior may be useful in reducing the impact of social anxiety on college students’ mobile phone addiction. Therefore, reinforcing the educational management of college students holds great practical significance.

### Social Anxiety and Mobile Phone Addiction

Social anxiety refers to the individuals’ experience of negative emotions such as worry, fear, and nervousness under social situations (Li et al., 2003). It is a psychosocial problem, and people with social anxiety prefer other forms of communication to face-to-face communication, especially through mobile technology devices, because a communication method not involving face-to-face communication can reduce anxiety (Darcin et al., 2016). Generally, social anxiety tends to persist from childhood to adolescence. A study reported that the social anxiety of college students is as high as 45.7% (Li et al., 2019). People with social anxiety are extremely afraid of real social situations and tend to be shy, nervous, embarrassed, afraid, and produce other negative emotions while communicating with others (Ashbaugh et al., 2010). Online communication can help people with social anxiety avoid the negative emotions and pressure produced during real communication, and these individuals feel more comfortable in online communication than in real communication (Prizant-Passal et al., 2016). According to the cognitive model of problematic Internet use, individuals experience interpersonal control in online communication, which is conducive to the alleviation of their negative emotions. Moreover, their Internet use behavior is strengthened by negative reinforcement, and they are prone to develop abstinence syndromes (Caplan, 2002). Lee et al. (2013) verified that the use of mobile media for social communication to escape social anxiety in real life is one of the reasons for the excessive use of social networks. Kim et al. (2006) demonstrated that people with a high social anxiety level are more likely to exhibit Internet addiction. Therefore, we hypothesize that college students’ social anxiety has a significant predictive effect on mobile phone addiction (H1).

### Social Anxiety, Regulatory Emotional Self-Efficacy, and Mobile Phone Addiction

Regulatory emotional efficacy represents a confidence level through which individuals can regulate their emotional state, and self-confidence is a manifestation of high self-efficacy (Bandura et al., 2003). It involves the perceived self-efficacy in expressing the positive affect, managing anger and irritation, and managing despondency and distress (Caprara et al., 2008). Kashdan and McKnight (2010) found that some individuals with social anxiety have poor emotion regulation ability, face difficulty in adapting to environmental changes, and experience varied negative emotions and strong hostile impulses. Individuals with social anxiety are emotionally instable, impatient, and impulsive; they express their emotions through repression and concealing and exhibit poor emotion regulation ability (Kashdan et al., 2011). In addition, the self-negative belief held by socially anxious individuals has an influence on their meta-evaluation of mood; therefore, their regulatory emotional self-efficacy is lower than that of other individuals (Mennin et al., 2005; Bassi et al., 2018). Kashdan and Roberts (2004) found a negative correlation between social anxiety and self-efficacy. Individuals with a high social anxiety level experience stronger negative effects and worse self-efficacy than those with a low social anxiety level. Previous studies have shown that regulatory emotional self-efficacy exerts an impact on individuals’ psychosocial adaptation and that it can effectively regulate externalized problems (such as aggression and crime) and internalized problems (such as depression and shyness) (Caprara et al., 2010). Furthermore, studies have demonstrated that regulatory emotional self-efficacy has not only a short-term influence on depression and aggression but also a long-term effect (Caprara et al., 2002, 2010). Therefore, college students’ social anxiety possibly exerts an influence on regulatory emotional self-efficacy and then leads to mobile phone addiction. Based on this assumption, we hypothesize that regulatory emotional self-efficacy plays a role in mediating the impact of college students’ social anxiety on their mobile phone addiction behavior (H2).

### Social Anxiety, Subjective Well-Being, and Mobile Phone

Subjective well-being represents an individual’s subjective evaluation and experience of life quality, and it serves as a significant psychological indicator that reflects the quality of life of an individual under a particular social situation (Diener et al., 1999). It is assessed according to the health condition, life, work, and interpersonal relationships (Diener and Ryan, 2009). Social anxiety is a crucial factor that affects individuals’ subjective well-being. It can substantially reduce individuals’ social motivations and the sense of pleasure generated by social communication, thereby reducing their perception of happiness (Li et al., 2017). People with social anxiety generally resort to negative means such as self-comfort, relying on others, fantasy, and exhibiting forgetfulness to avoid problems and relieve anxiety and tension (Li et al., 2003). However, social relations can exert a strong impact on subjective well-being. Individuals’ life satisfaction is increased once their social needs are met (Leung et al., 2011). Ye et al. (2021) observed a significant negative correlation between social anxiety and subjective well-being of college students. The authors attached great importance to the social skills and interpersonal relationships among college students, whereas the emergence of social anxiety was reported to reduce the students’ overall well-being. Another study on college students with different anxiety levels showed that the higher the level of social anxiety, the lower is the subjective well-being, and the lower the social anxiety level, the higher is the subjective well-being (Öztürk and Mutlu, 2010). Social anxiety is a key factor that influences individuals’ subjective well-being, which in turn can significantly reduce the motivation of individual social behavior and the sense of pleasure derived from social communication, thereby reducing the individual perception of happiness.

Previous studies have found that subjective well-being has a significant impact on both physical and mental health of individuals and their Internet addiction behavior (Li et al., 2017). According to the compensation theory of network satisfaction, the unmet psychological needs in realities strongly influence the college students’ Internet addiction behavior through network satisfaction compensation (Parker and Plank, 2000). Empirical research has demonstrated a significant negative correlation between subjective well-being and dependence on mobile phone; the higher the subjective well-being, the lower is the mobile phone addiction (Deng et al., 2015). A high level of subjective well-being serves as a protective factor for Internet addiction (Durak, 2011; Bozoglan et al., 2013). Therefore, social anxiety has an impact on subjective well-being, which in turn has an influence on addiction behavior. Thus, it is reasonable to predict that subjective well-being plays a mediating role in the relation between social anxiety and mobile phone addiction among college students. Based on this assumption, we propose the third hypothesis that subjective well-being plays a mediating role in the impact of social anxiety on mobile phone addiction among college students (H3).

### Social Anxiety, Regulatory Emotional Self-Efficacy, Subjective Well-Being, and Mobile Phone Addiction

Although social anxiety, as a type of negative emotion, has a negative impact on subjective well-being, Tsapelas et al. (2010) described regulatory emotional self-efficacy as a significant predictor of subjective well-being. Emotion regulation theory has been put forward to identify factors that affect the relation between emotions and behaviors; among these, self-efficacy is a key regulating factor (Heuven et al., 2006). Caprara et al. (2002) found that the differences in emotion regulation among individuals depend on the belief in their own emotion regulation ability, whereas such belief has an impact on the actual effect of individuals’ emotion regulation. As a type of self-efficacy, regulatory emotional self-efficacy not only has a direct impact on behaviors but also can indirectly affect behaviors by influencing cognition, motivations, decisions, and emotions, thereby playing a vital role in regulating individual personality and behavior and maintaining the individual mental health level (Tang et al., 2010). Research suggests that regulatory emotional self-efficacy has an impact on the individuals’ evaluation of life quality, that is, their subjective well-being. It is mainly reflected in the fact that the individuals having a high level of regulatory emotional self-efficacy are capable of effectively dealing with their emotions and timely adjust and control their negative emotions, thus maintaining a positive attitude toward things and exhibiting higher subjective well-being (Caprara and Steca, 2006). Moreover, the level of negative indicators such as depressive symptoms that reflect well-being from the negative aspect will be lower (Au et al., 2009). Other studies have found that individuals with a high self-efficacy exhibit stronger well-being experience (Cohen and Cairns, 2012). A study on the three fields of self-efficacy (academic, social, and emotional) demonstrated that regulatory emotional self-efficacy is a key factor for the well-being of adolescents (Andretta and McKay, 2020). The aforementioned findings suggest that social anxiety may lead to the reduction of regulatory emotional self-efficacy, and subjective well-being is conducive to the reduction of mobile phone addiction among college students. Therefore, it is reasonable to predict that the decrease in social anxiety among college students may be associated with better regulatory emotional self-efficacy, which in turn exert a positive impact on subjective well-being, and the improved subjective well-being may help in reducing mobile phone addiction among college students. Based on this assumption, we put forward the fourth hypothesis that both regulatory emotional self-efficacy and subjective well-being play a chain mediating role in the influence of social anxiety on mobile phone addiction of college students (H4).

## Materials and Methods

### Participants

Using the convenient sampling method, we conducted a questionnaire survey on students at a university in Hebei Province, China, on an online questionnaire platform. In September 2021, teachers at the administration office sent the questionnaires in electronic forms to the students at our request. It took a university student approximately 30 min to fill in the questionnaire. We distributed 735 questionnaires and received 680 valid ones after eliminating invalid ones, with a 92.5% response rate. Among the students who returned the valid questionnaires, 450 were female and 230 were male; 216 were freshmen, 188 were sophomores, 174 were juniors, and 100 were seniors (see Table 1 for details). According to the formula provided by Israel (1992) for calculating the sample size, that is, sample size = z^2^ × p(1−p)/e^2^/1 + (z^2^ × p(1−p)/e^2^N), *z* = 2.58, *p* = 0.5, *N* = 3,000, and *e*^2^ = 0.0025, the formal sample size should be no less than 545. The size of our study met the sampling criteria. The study was conducted in accordance with the Declaration of Helsinki and all subjects were willing to cooperate and had signed informed consent forms. We gave due consideration to their privacy and wishes and informed them that they could refuse to participate or withdraw from the study at any time (Goodyear et al., 2007). The study was also approved by the Ethics Committee of Hengshui College, China.

**TABLE 1:** Background variable characteristics.

Variables	Grouping	Percentages
Gender	Male	33.8%
	Female	66.2%
College grade	Freshman	31.8%
	Sophomore	27.6%
	Junior	25.6%
	Senior	14.7%

### Measurement

#### Social Anxiety

The social anxiety scale of college students compiled by Qian et al. (2005) was used to assess the social anxiety level; the scale comprises 22 items from three dimensions, namely tension and anxiety, social sensitivity, and social confidence; among these, the third dimension involves a reverse scoring question pattern. The five-point scoring system, from 1 = “Completely do not conform” to 5 = “Completely conform,” was adopted. The higher the score, the higher is the individual social anxiety level. The Cronbach α value of the scale in the present study was 0.946. The result of confirmatory factor analysis indicated that the χ^2^/df ratio was 5.990. This ratio is susceptible to the sample size; thus, when it is greater than 3, other fitness indicators should be determined (Wheaton, 1987). Regarding other fitness indicators, RMSEA = 0.08 (lower than the standard value of 0.1); SRMR = 0.051 (lower than the standard value of 0.8) (MacCallum et al., 1992); CFI = 0.898; NFI = 0.880; GFI = 0.859; and TLI = 0.884, and the fitting indices of the four variables were greater than 0.8, which indicated that the model fitted well (Bollen, 1989; Gefen et al., 2000; Schumacker et al., 2004).

#### Regulatory Emotional Self-Efficacy

The regulatory emotional self-efficacy scale compiled by Caprara et al. (2010) was adopted. The scale was first translated into Chinese language by Wen et al. (2009) to conduct tests in college students; the results proved the reliability and validity of the scale. The scale comprises 12 items from three dimensions, namely the perceived self-efficacy in expressing positive affect, the perceived self-efficacy in regulating despondency and distress, and the perceived self-efficacy in regulating anger and irritation. The five-point scoring system, from 1 = “Completely do not conform” to 5 = “Completely conform” was adopted. The total score of each item was summed up to obtain the total score for regulatory emotional self-efficacy. A high score denoted the high individual regulatory emotional self-efficacy. The Cronbach α value of the scale in the present study was 0.869. The results of confirmatory factor analysis are as follows: χ^2^/df = 4.008, RMSEA = 0.067, SRMR = 0.064, CFI = 0.961, NFI = 0.949, GFI = 0.953, and TLI = 0.950, indicating that the model fitted well.

#### Subjective Well-Being

The subjective well-being scale compiled by Pontin et al. (2013) was adopted. The scale comprises 24 items from three dimensions, namely psychological well-being, physical well-being, and interpersonal relationship well-being. An item “Are you satisfied with your sexual life?” was deemed unsuitable for college students and hence excluded. Altun et al. (2014) proved the reliability and validity of the scale for college students. The five-point scoring system, from 1 = “Never have” to 5 = “Always have,” was used. The Cronbach α value of the scale in the present study was 0.950. The results of confirmatory factor analysis are as follows: χ^2^/df = 5.430, RMSEA = 0.081, SRMR = 0.039, CFI = 0.929, NFI = 0.914, GFI = 0.888, and TLI = 0.917, indicating that the model fitted well.

#### Mobile Phone Addiction

The mobile phone addiction scale compiled by Leung (2008) was adopted. The scale comprises 17 items from four dimensions, namely uncontrolled addiction, abstinent addiction, avoidant addiction, and inefficient addiction. The five-point scoring system, from 1 = “Never have” to 5 = “Always have,” was adopted. The total score of each item was summed up to obtain the total score for mobile phone addiction. The higher the score, the more severe is the mobile phone addiction. The scale has displayed good reliability and validity in previous studies (Lian et al., 2016). The Cronbach α value of the scale in the present study was 0.945. The results of confirmatory factor analysis are as follows: χ^2^/df = 8.887, RMSEA = 0.108, SRMR = 0.046, CFI = 0.937, NFI = 0.929, GFI = 0.883, and TLI = 0.916, indicating that the model fitted well.

#### Data Analysis

SPSS and AMOS were used to manage and analyze the data; SPSS was used mainly for preliminary data processing, descriptive statistics, reliability and validity tests, and correlation analysis, whereas AMOS was used for confirmatory factor analysis and mediating effect analysis through the structural equation model of latent variables. Harman single-factor test was performed in SPSS to determine the influence of common method variance on the study results. Furthermore, we conducted a test on the analysis result of non-rotating factor by including the social anxiety level, regulatory emotional self-efficacy, subjective well-being, and mobile phone addiction in the exploratory factor analysis. The results showed that a total of 11 factors exhibited characteristic roots greater than 1, with the variance interpretation rate of the first factor being 38.6% (less than 50%) (Harris et al., 2009), which indicated that the common method variance has a slight impact on the present study results.

## Results

### Descriptive Statistics and the Correlation Among the Studied Variables

Table 2 shows the descriptive statistics of each studied variable including the mean value, standard deviation, and correlation strength among the variables. The correlation coefficients between the studies variables ranged from 0.21 to 0.75, all of which exhibited a significance level of *p* < 0.001. Therefore, no collinearity problem was evident.

**TABLE 2:** Descriptive statistics and correlation between observed variables.

Variables	M	SD	TA	SIC	SIS	POS	DES	ANG	PSY	PHY	INT	UNC	ABS	AVO	INE
TA	2.59	0.86	−												
*SIC*	2.62	0.70	0.65	−											
SIS	2.57	0.83	0.75	0.60	−										
POS	4.02	0.68	−0.26	−0.40	−0.25	−									
DES	3.46	0.75	−0.51	−0.54	−0.53	0.32	−								
ANG	3.35	0.78	−0.41	−0.45	−0.49	0.21	0.68	−							
PSY	3.63	0.80	−0.52	−0.55	−0.56	0.43	0.60	0.52	−						
PHY	3.71	0.82	−0.45	−0.47	−0.50	0.27	0.54	0.49	0.75	−					
INT	3.79	0.83	−0.46	−0.48	−0.47	0.33	0.45	0.37	0.68	0.67	−				
UNC	2.12	0.97	0.53	0.56	0.56	−0.35	−0.52	−0.50	−0.63	−0.59	−0.57	−			
ABS	2.27	1.12	0.56	0.57	0.57	−0.30	−0.54	−0.48	−0.57	−0.55	−0.52	0.74	−		
AVO	2.76	1.17	0.44	0.49	0.49	−0.22	−0.45	−0.45	−0.44	−0.42	−0.37	0.59	0.63	−	
INE	2.60	1.10	0.56	0.54	0.51	−0.27	−0.52	−0.50	−0.50	−0.53	−0.50	0.73	0.68	0.58	−

*The correlation coefficients all reached significant levels (p < 0.001). M, mean; SD, standard deviation; TA, tension and anxiety; SIC, social interaction confidence; SIS, social interaction sensitivity; POS, positive affect; DES, despondency and distress; ANG, anger and irritation; PSY, psychological well-being; PHY, physical well-being; INT, interpersonal relationship well-being; UNC, uncontrolled addiction; ABS, abstinent addiction; AVO, avoidant addiction; INE, inefficient addiction.*

In this questionnaire, the skewness absolute values for the 74 items were between 0.028 and 0.875, and the kurtosis absolute values for the 74 items were between 0.01 and 1.156 (see Supplementary Appendix for details). The results satisfied the standards of the absolute value for skewness < 2 and kurtosis < 7 (Curran et al., 1996). The research data met normal distribution.

### Structural Model

In this research, *t*-test and ANOVA analysis result show that gender (*t* = −0.761, *p* > 0.050) and college grade (*F* = 1.109, *p* > 0.050) have no significant difference in college students’ mobile phone addiction. Therefore, this research does not control the impact on college students’ mobile phone addiction of gender and college grade.

**FIGURE 1:**
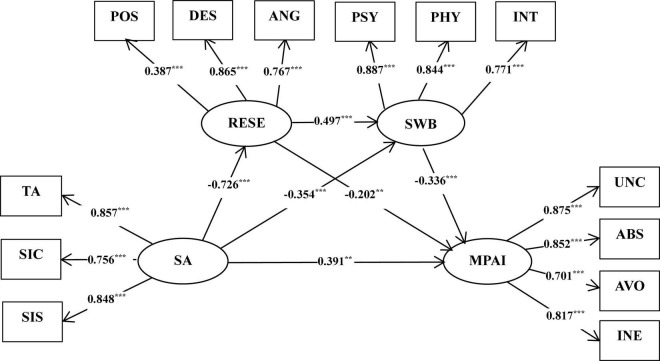
Final standardized parameter values of the mode. SA, social anxiety; RESE, regulatory emotional self-efficacy; SWB, subjective well-being; MPAI, mobile phone addiction; TA, tension and anxiety; *SIC*, social interaction confidence; SIS, social interaction sensitivity; POS, positive affect; DES, despondency and distress; ANG, anger and irritation; PSY, psychological well-being; PHY, physical well-being; INT, interpersonal relationship well-being; UNC, uncontrolled addiction; ABS, abstinent addiction; AVO, avoidant addiction; INE, inefficient addiction. ***p < 0.001, **p < 0.01.

A structural model was used to examine the relationship among social anxiety, regulatory emotional self-efficacy, subjective well-being, and mobile phone addiction, which showed that χ^2^/df = 5.201, RMSEA = 0.079, SRMR = 0.040, CFI = 0.956, NFI = 0.946, GFI = 0.935, and TLI = 0.942, indicating that the model fitted well. The results are shown in Figure 1. First, social anxiety was found to have a significant positive impact on mobile phone addiction (β = 0.391, *p* < 0.01), thus verifying H1. Second, social anxiety was found to have a significant negative impact on regulatory emotional self-efficacy (β = −0.726, *p* < 0.001), whereas regulatory emotional self-efficacy was found to have a significant negative influence on mobile phone addiction (β = −0.202, *p* < 0.01). This finding indicated that social anxiety may indirectly affect mobile phone addiction by virtue of regulatory emotional self-efficacy. In addition, social anxiety was found to have a significant negative impact on subjective well-being (β = −0.354, *p* < 0.001), whereas subjective well-being was found to have a significant negative impact on mobile phone addiction (β = −0.336, *p* < 0.001). The finding suggested that social anxiety affects mobile phone addiction possibly by affecting subjective well-being. Finally, regulatory emotional self-efficacy was found to significantly and positively affect subjective well-being (β = 0.497, *p* < 0.01), indicating that social anxiety influences subjective well-being possibly by affecting regulatory emotional self-efficacy and finally affects mobile phone addiction.

In the present study, the deviation correction percentile Bootstrap method (repeated the sampling for 500 times) was used to determine the mediating effect, and the confidence interval was set at 95%. Exclusion of 0 in the confidence interval indicates a significant mediating effect (Hayes, 2013). The results are shown in Table 3. Regulatory emotional self-efficacy was found to play a significant mediating role in the relation between social anxiety and mobile phone addiction, indicating that H2 is tenable. Furthermore, subjective well-being had a significant mediating impact on the relation between social anxiety and mobile phone addiction, indicating that H3 is tenable. In addition, both regulatory emotional self-efficacy and subjective well-being were found to play a significant chain mediating role in the relation between social anxiety and mobile phone addiction, thereby verifying H4.

**TABLE 3:** Mediation effect with bootstrapping.

Path	Effect value	Proportion	95% confidence interval	
			Lower limit	Upper limit
SA → RESE → MPAI	(−0.726) × (−0.202) = 0.147**	0.147/0.778 = 0.189	−0. 009	−0. 308
SA → SWB → MPAI	(−0.354) × (−0.336) = 0.119***	0.119/0.778 = 0.153	−0. 039	−0. 232
SA → RESE → SWB → MPAI	(−0.726)×0.497 × (−0.336) = 0.121***	0.121/0.778 = 0.156	−0. 049	−0. 234
Direct effect (SA → MPAI)	0.391**	0.391/0.778 = 0.503	−	−
Total mediation	0.387	0.387/0.778 = 0.497	−	−
Total effect	0.778	−	−	−

The effect values in the table are standardized parameter values.**p < 0.01, ***p < 0.001. SA, social anxiety; RESE, regulatory emotional self-efficacy; SWB, subjective well-being; MPAI, mobile phone addiction.

## Discussion

Based on the previous studies, we developed a chain mediating effect model, with social anxiety as the predictive variable, regulatory emotional self-efficacy and subjective well-being as the mediating variables, and mobile phone addiction as the outcome variable. The results of our study supported the self-efficacy theory, which suggests that regulatory emotional self-efficacy, as a type of self-efficacy, both directly and indirectly influences behaviors by influencing cognition, motivations, decisions, and emotions (Caprara and Steca, 2006; Caprara et al., 2008).

The present study indicated that college students’ social anxiety has a significant positive impact on their mobile phone addiction; the finding is consistent with those of previous studies (Kim and Koh, 2018; Kong et al., 2020). Kardefelt-Winther (2014) proposed the compensatory Internet use theory, while considering the fact that various needs of adolescents are driven by individual development needs, and if the needs are hindered by interference factors, a “psychological compensation” process will occur. If the needs of adolescents cannot be met, a “pathological compensation” will occur, and they are likely to seek compensation through a way that is easy to produce a sense of achievement, such as playing games on the Internet. Social anxiety is characterized mainly by the fear of evaluation from other people, with individuals believing that other people will provide negative feedback, resulting in fear while communicating (Haxby et al., 2000). In the new social communication mode, college students can avoid face-to-face communication, thus reducing the possibility of cognitive processing deviation due to the negative expression of other people (Yoon et al., 2007). Therefore, students can communicate more comfortably using mobile phones and the Internet (Lee and Stapinski, 2012). People with high social anxiety tend to choose mobile phones or the Internet as substitutes for real social communication, eventually exhibiting high dependence on mobile phones and the Internet.

The present study results suggest that the regulatory emotional self-efficacy mediates the relation between social anxiety and mobile phone addiction of college students, consistent with the finding of previous studies indicating that regulatory emotional self-efficacy has a significant impact on mobile phone addiction (Mesurado and Malonda, 2018). Regulatory emotional self-efficacy plays a crucial role in maintaining mental health (Fredrickson et al., 2008). As a negative emotion, social anxiety influences the individual regulatory emotional self-efficacy (Sommers and Vodanovich, 2000). Individuals having a low regulatory emotional self-efficacy level tend to experience negative emotions for a long time, eventually exhibiting uncontrolled and aggressive or addictive behaviors (Liu et al., 2021). On the one hand, college students with a high social anxiety level lack social skills and use inappropriate coping strategies in social activities, leading to unfavorable social outcomes (Li et al., 2003) and thus addiction behaviors. On the other hand, college students with a high social anxiety level have poor emotion regulation ability and cognition, which render them incapable of adjusting their emotional state well (Kashdan et al., 2011), leading to the low regulatory emotional self-efficacy and thus mobile phone addiction.

Subjective well-being has a partial mediation effect on the relation between social anxiety and mobile phone addiction of college students, which is consistent with a study indicating that subjective well-being can significantly and negatively predict mobile phone addiction (Bozoglan et al., 2013). College students generally face problems in learning, interpersonal relationship, and emotions. In the face of learning pressure, coping with the pressure through positive means can improve individual subjective well-being, whereas the use of negative means can deteriorate individual subjective well-being (Karlsen et al., 2006). Individuals with strong subjective well-being exhibit better mental health, self-esteem, and self-control (Bozoglan et al., 2013), less negative emotional experience such as depression and anxiety, and strong adaptability (Klug and Maier, 2015). Such individuals can flexibly adjust to the interaction between themselves and surrounding environment; they not only achieve satisfaction in playing online games without becoming addicted but also display sufficient ability to deal with setbacks in life and achieve satisfaction in real life (Mascia et al., 2020).

College students’ social anxiety exerts an impact on mobile phone addiction possibly through the mediation of regulatory emotional self-efficacy and subjective well-being, which is an important means by which social anxiety affects mobile phone addiction in college students. This finding is consistent with those of previous studies suggesting that regulatory emotional self-efficacy can significantly and positively affect subjective well-being (Caprara and Steca, 2006; Tsapelas et al., 2010). High regulatory emotional self-efficacy is conducive to the improvement in interpersonal relationship efficacy, which in turn can facilitate the formation of satisfactory and harmonious interpersonal relationships, improve subjective well-being, and enable individuals to effectively cope with addiction behaviors (Tang et al., 2010). College students with social anxiety generally tend to lose control of their emotions and lack confidence in effectively regulating their emotions, leading to more negative emotional experience, decreased well-being, and mobile phone addiction (Bozoglan et al., 2013). College students with high regulatory emotional self-efficacy can effectively communicate with others. In the presence of a negative stimulus, these individuals take effective measures, for example, by changing view of events to eliminate inside depression, and display strong confidence in effectively regulating their emotions. Therefore, they experience fewer negative emotions and reduced risk of developing mobile phone addiction (Caprara et al., 2002; Durak, 2011).

### Practical Significance

The present study provides ideas for the prevention of mobile phone addiction among college students. First, social anxiety can directly and significantly predict mobile phone addiction. Therefore, colleges should pay more attention to the social anxiety of students, and reduce their social anxiety by organizing various activities and encouraging students to actively participate in those activities. Second, social anxiety can affect mobile phone addiction through chain mediation between regulatory emotional self-efficacy and subjective well-being, indicating that both these variables are the key factors influencing college students’ mobile phone addiction. Thus, colleges should attach importance to the cultivation of regulatory emotional self-efficacy and subjective well-being of students. In practical college education, emotion education courses or activities should be considered as the carrier to transfer emotion regulation knowledge and skills to college students and guide them to effectively regulate their emotions to gradually improve their regulatory emotional self-efficacy. The improvement of regulatory emotional self-efficacy is not only conducive to the improvement of students’ emotional state but also essential for students to gain a sense of control in daily emotional experience and thus enhance their self-confidence.

### Limitations and Future Research Directions

The present study has some limitations. First, the study design was cross-sectional. Although previous studies have provided the basis for such studies, establishing a precise causal relationship using this method is challenging. Therefore, longitudinal studies are warranted to ascertain the causal relationship between social anxiety and mobile phone addiction. Second, the study adopted the questionnaire method; thus, future studies should adopt a qualitative or quasi-experimental design to further explore the possible causes of mobile phone addiction.

## Data Availability Statement

The raw data supporting the conclusions of this article will be made available by the authors, without undue reservation.

## Ethics Statement

The studies involving human participants were reviewed and approved by the Hengshui University. The patients/participants provided their written informed consent to participate in this study. Written informed consent was obtained from the individual(s) for the publication of any potentially identifiable images or data included in this article.

## Author Contributions

ZX designed the study, analyzed the data, and drafted the manuscript. JH assisted in analyzing and interpreting the data and participated in the revision of the manuscript. Both authors contributed to the study and approved the submitted version.

## Conflict of Interest

The authors declare that the research was conducted in the absence of any commercial or financial relationships that could be construed as a potential conflict of interest.

## Publisher’s Note

All claims expressed in this article are solely those of the authors and do not necessarily represent those of their affiliated organizations, or those of the publisher, the editors and the reviewers. Any product that may be evaluated in this article, or claim that may be made by its manufacturer, is not guaranteed or endorsed by the publisher.
