# Supplementary material for: The Relation Between College Students’ Social Anxiety and Mobile Phone Addiction: The Mediating Role of Regulatory Emotional Self-Efficacy and Subjective Well-Being
Source: Front Psychol. 2022 May 2;13:861527. doi: 10.3389/fpsyg.2022.861527 (PMC9108388; doi:10.3389/fpsyg.2022.861527)
Supplement: Supplementary file 1 [file Table_1.pdf]

Appendix:

Items	Mean ± SD	Normal distribution	
		Skewness	Kurtosis
**Social anxiety**			
TA_1: I am afraid of failing to express the meaning when talking to other people.	2.53±1.102	-.003	-.891
TA_2: l often get nervous when talking to the opposite sex.	2.65±1.158	.099	-.780
TA_3, Even though I knew I should not be nervous at a social event, I could not control myself.	2.89±1.129	-.130	-.743
TA_4, I get nervous for a period before going to social activities.	2.99±1.161	-.181	-.745
TA_5, I worry about blushing when interacting with other people.	2.33±1.098	.432	-.597
TA_6, I am always worried about misbehaving when dealing with people.	2.94±1.130	-.173	-.704
TA_7, As long as anyone says I'm not good, I will be sad for a long time.	2.58±1.122	.284	-.610
TA_8, I often get nervous and sweat when interacting with certain people (boss, opposite sex).	2.50±1.106	.288	-.617
TA_9, I could not relax at a social event; even I had tried my best.	2.27±0.987	.569	.073
TA_{10}, I am afraid to look people in the eyes when talking.	2.17±1.006	.530	-.352
SIC_1, Even though the room is full of strangers, I will go in if I need to.	2.21±0.989	.708	.097
SIC_2, After a special relationship with people, l believe that others will like me.	2.80±0.968	.097	.037
SIC_3, I do not miss the chance to make new friends.	2.64±0.983	.022	-.333
SIC_4, I am delighted with how l deal with people.	2.73±0.877	.056	.379
SIC_5, l usually like to communicate with others.	2.66±0.899	.107	.159
SIC_6, I have confidence in my ability to interact with people.	2.69±0.960	.109	-.155
SIS_1, I do not think people will have a good impression of me.	2.26±0.928	.384	.011
SIS_2, After attending social events, I always feel like I am not doing well at the time.	2.79±1.008	-.103	-.500
SIS_3, l feel that if I misbehave with others, others will look down on me.	2.34±1.015	.411	-.323
SIS_4, When I interact with the opposite sex, l often worry that others will think badly of me.	2.39±1.059	.374	-.604
SIS_5, I always worry about losing face in front of people.	2.91±1.109	-.098	-.676
SIS_6, I'm always worried that people will think I have a poor performance when interacting with people.	2.71±1.031	.159	-.470
**Regulatory emotional self-efficacy**			
POS_1, Express joy when good things happen to you?	4.09±0.831	-1.131	2.116
POS_2, Feel gratified over achieving what you set out to do?	4.20±0.749	-1.062	2.226
POS_3, Rejoice over your successes?	4.14±0.844	-1.027	1.477
POS_4, Express enjoyment freely at parties?	3.67±0.897	-.253	-.121
DES_1, Keep from getting dejected when you are lonely?	3.32±0.958	-.208	-.205
DES_2, Keep from getting discouraged by strong criticism?	3.39±0.938	-.178	-.275
DES_3, Reduce your upset when you don't get the appreciation you feel you deserve?	3.45±0.872	-.198	-.120
DES_4, Keep from getting discouraged in the face of difficulties?	3.68±0.862	-.440	.252
ANG_1, Manage negative feelings when reprimanded by your parents or significant others?	3.36±0.929	-.232	-.192
ANG_2, Avoid getting upset when others keep giving you a hard time?	3.16±1.017	-.154	-.402
ANG_3, Get over irritation quickly for wrongs you have experienced?	3.37±0.907	-.300	.244
ANG_4, Avoid flying off the handle when you get angry?	3.50±0.913	-.291	.016
**Subjective well-being**			
PSY_1, Do you feel depressed or anxious?	2.32±0.957	-.898	.352
PSY_2, Do you feel able to enjoy life?	3.68±0.949	-.635	.249
PSY_3, Do you feel you have a purpose in life?	3.86±1.093	-.385	-.398
PSY_4, Do you feel optimistic about the future?	3.79±1.074	-.696	-.089
PSY_5, Do you feel in control of your life?	3.46±1.039	-.364	-.367
PSY_6, Do you feel happy with yourself as a person?	3.88±1.007	-.816	.328
PSY_7, Are you happy with your looks and appearance?	3.78±1.063	-.169	-.532
PSY_8, Do you feel able to live your life the way you want?	3.59±1.042	-.338	-.477
PSY_9, Are you confident in your own opinions and beliefs?	3.80±0.974	-.577	.035
PSY_{10}, Do you feel able to do the things you choose to do?	3.88±0.943	-.526	.199
PSY_{11}, Do you feel able to grow and develop as a person?	3.92±0.958	-.802	.479
PSY_{12}, Are you happy with yourself and your achievements?	3.59±1.023	-.021	-.541

PHY_1. Are you happy with your physical health?	3.78±1.036	-.712	.022
PHY_2. Are you happy with the quality of your sleep?	3.70±1.092	-.737	-.032
PHY_3. Are you happy with your ability to perform daily living activities?	3.95±1.000	-.970	.730
PHY_4. Are you happy that you have enough money to meet your needs?	3.90±1.016	-.823	.369
PHY_5. Are you happy with your opportunity for exercise/ leisure?	3.52±1.126	-.596	-.361
PHY_6. Are you happy with access to health services?	3.73±1.057	-.609	-.218
PHY_7. Are you happy with your ability to work?	3.42±0.957	-.319	.040
INT_1. Are you happy with your personal and family life?	3.81±1.033	-1.371	1.391
INT_2. Are you happy with your friendships and personal relationships?	3.99±0.968	-.946	.764
INT_3. Are you comfortable about way you relate and connect with others?	3.80±0.955	-.635	.308
INT_4. Are you able to ask someone for help with a problem?	3.56±0.980	-.325	-.162
**Mobile phone addiction**			
UNC_1. Your friends and family complained about your use of the mobile phone.	2.44±1.135	.336	-.691
UNC_2. You have been told that you spend too much time on your mobile phone.	2.40±1.156	.493	-.580
UNC_3. You have tried to hide from others how much time you spend on your mobile phone.	1.90±1.175	1.102	.134
UNC_4. You have received mobile phone bills you could not afford to pay.	1.56±1.085	1.992	2.575
UNC_5. You find yourself engaged on the mobile phone for longer period of time than intended.	2.26±1.263	.071	-1.024
UNC_6. You have attempted to spend less time on your mobile phone but are unable to.	2.01±1.203	.405	-.787
UNC_7. You can never spend enough time on your mobile phone.	2.33±1.213	.534	-.656
ABS_1. When out of range for some time, you become preoccupied with the thought of missing a call.	2.22±1.229	.587	-.846
ABS_2. You find it difficult to switch off your mobile phone.	2.54±1.428	.355	-1.273
ABS_3. You feel anxious if you have not checked for messages or switched on your mobile phone for some time.	2.14±1.243	.816	-.447
ABS_4. You feel lost without your mobile phone.	2.18±1.247	.754	-.525
ABS_5. If you don't have a mobile phone, your friends would find it hard to get in touch with you.	2.29±1.239	-.009	-.959
AVO_1. You have used your mobile phone to talk to others when you were feeling isolated.	2.71±1.257	.125	-1.022
AVO_2. You have used your mobile phone to talk to others when you were feeling lonely.	2.81±1.220	.063	-.950
AVO_3. You have used your mobile phone to make yourself feel better when you were feeling down.	2.76±1.183	-.044	-.821
INE_1. You find yourself occupied on your mobile phone when you should be doing other things, and it causes a problem.	2.57±1.153	.232	-.743
INE_2. Your productivity has decreased as a direct result of the time you spend on the mobile phone.	2.64±1.155	.169	-.776

Note: TA, tension and anxiety; SIC, social interaction confidence; SIS, social interaction sensitivity; POS, positive affect; DES, despondency and distress; ANG, anger and irritation; PSY, psychological well-being; PHY, physical well-being; INT, interpersonal relationship well-being; UNC, uncontrolled addiction; ABS, abstinent addiction; AVO, avoidant addiction; INE, inefficient addiction.
